# Adolescent and nurse perspectives of psychotherapeutic interventions for PTSD delivered through task-shifting in a low resource setting

**DOI:** 10.1371/journal.pone.0199816

**Published:** 2018-07-10

**Authors:** Tanya van de Water, Jaco Rossouw, Elna Yadin, Soraya Seedat

**Affiliations:** 1 Department of Psychiatry, Stellenbosch University, Cape Town, South Africa; 2 Department of Psychiatry, University of Pennsylvania, Pennsylvania, United States of America; The Ohio State University, UNITED STATES

## Abstract

**Background:**

This investigation compared the perceived effectiveness of supportive counselling (SC) and prolonged exposure for adolescents (PE-A) by treatment users (adolescents with PTSD) and non-specialist treatment providers (supervised nurses).

**Method:**

Adolescent participants and nurse providers were purposively recruited to share their experiences of trial participation through face to face semi-structured in-depth interviews and treatment-specific focus groups (all recorded). Twelve adolescent participant transcripts (ten interviews and two focus groups) and three nurse provider transcripts were doubly transcribed. Thematic content analysis was applied using Atlas.ti software. Two emerging themes are presented in this paper: 1) Perceptions of the intervention and 2) Usefulness of the intervention.

**Results:**

Regardless of treatment arm, adolescents experienced warm counselling relationships and described the process of extending trust to the counselor. Adolescents in the PE-A arm provided clear descriptions of session structure and treatment rationale compared with adolescents receiving SC. The most helpful tools were breathing retraining and imaginal exposure for PE-A and creation of distraction strategies during non-directive SC. Adolescents in both arms continued to use the techniques acquired during treatment and reported symptom improvement. Participants who received SC acknowledged ongoing reexperiencing. Nurses perceived SC to be an immediately transferable skill, but feedback on their preference for one intervention over the other was inconclusive.

**Conclusion:**

Both PTSD treatment strategies, implemented by non-specialists, were perceived as helpful. Overall, adolescents reported warm therapeutic relationships and a reduction in PTSD symptoms. Nurses stated that they would require institutional support to ensure delivery of these interventions in a scalable and sustainable manner.

## Introduction

South Africa shares with its neighboring sub-Saharan low and middle-income countries (LMICs), a struggle to attain universal and equitable access to health care [1]. Mental health specialists trained in evidence-based psychosocial treatments (EBTs) of common mental disorder are scarce in South Africa [2] and are mostly based at tertiary hospitals.

Task-shifting, the redistribution of tasks from specialists to those with abbreviated training, is increasingly offered as a solution to the shortage of treatment providers in order to make mental healthcare more accessible [3]. Recent randomized controlled trials (RCTs) conducted in sub-Saharan Africa provided promising results in reducing trauma symptoms amongst children and adolescents diagnosed with PTSD [4, 5]. These RCTS utilised Trauma-Focussed Cognitive Behavioural Therapy and Prolonged Exposure for Adolescents (TF-CBT and PE-A) and were task-shifted to lay-counsellors and non-specialist health workers (nurses), respectively. Recent studies on treatment of PTSD in adults in other LMIC settings also reported encouraging results. These include treatment in adult Ugandan refugees using narrative exposure therapy and trauma counselling [6]; in adult survivors of systematic violence in Thailand and Iraq using Common Elements Treatment Approach (CETA) [7]; and, in adult survivors of torture in Iraq using Cognitive Processing Therapy and CETA in community settings [8]. Several recent studies examined the contribution of non-trauma-focussed treatments in comparison to traum-focussed approaches on symptoms of anxiety, depression, and levels of functioning. A problem-solving therapy approach showed promising results when compared to a modified trauma-focussed intervention in treating civil-conflict and disaster-affected children in Indonesia [9]. Another RCT study from the same area, also using problem solving therapy focussing on the treatment of war-affected adults, did not find any significant improvement in anxiety and depression symptoms [10]. A non-trauma based intervention (Child Friendly Spaces) with Congolese youth affected by war achieved significant improvement in PTSD symptoms [11]. An RCT conducted in Iraq with survivors of torture and related trauma comparing a support, skills and psychoeducation program with a waitlist control did not provide significant improvement in trauma symptoms [12].

The debate about implementing EBTs in LMIC is ongoing [13]. Dissemination and implementation of EBTs for routine use is deemed to be the next step [14]. The challenges identified [14] include lack of adequately trained personnel; attrition of treatment providing personnel; danger and instability in the countries or areas of implementation; scarcity of facilities at which to provide treatment; lack of transportation infrastructure; stigma associated with accessing treatment; instability of leadership within public health systems as a result of high turnover in personnel and instability in health systems; lack of trust by users of the health system: policy-related low priority of mental health treatment; and insufficient funding to sustain implemented interventions.

Integrating mental healthcare within primary healthcare clinics is offered as a possible solution to the lack of access to mental healthcare [13]. It is argued that in order to build primary mental healthcare systems, all stakeholders (including policy makers, mental health specialists, non-specialist community health workers and community members) will have to be involved. Mental health specialists will have to redefine their roles and be actively involved in training and supervising non-specialist health workers in psychosocial treatments [13, 15]. Defining a role for non-specialist heath workers involved in task-sharing and appropriate compensation for performing these new mental health roles [15] will be central to sustained implementation. Identifying the perceptions and experiences of treatment users and providers of the treatments will support appropriate implementation of task-shifted EBTs within community settings [16].

A recent RCT aimed at studying the comparative effectiveness of two active treatments in reducing PTSD and other associated symptoms was conducted in Cape Town, South Africa using a task-shifting approach, in which nurses trained and supervised for this study provided two community-based psychological treatments for adolescents suffering from PTSD [17].

### Prolonged exposure for adolescents

Some PTSD treatment guidelines recommend the use of trauma focused treatment that includes an exposure element [18–21]. Prolonged exposure therapy is a trauma focused treatment initially designed for adults [22–24]. It has been shown to be effective within a community setting [25] and meets the requirements for dissemination of PTSD interventions in LMICs, as suggested by some researchers in the field [26]. Similarly, the adolescent adaptation of this intervention, prolonged exposure treatment for adolescents (PE-A) has been successfully implemented in community settings in the USA [27], Israel [28], and in an exploratory pilot study in South Africa [6]. Treatment comprises of eight modules that expound on the treatment rationale, breathing retraining, common reactions to trauma, in-vivo exposure using in-vivo hierarchy, and imaginal exposure (emotional processing of the traumatic memory through retelling) [6].

Although a highly efficacious treatment for PTSD [24], prolonged exposure therapy is underutilized [29]. Apart from a shortage of adequately trained professionals, treatments that include an element of exposure are sometimes perceived as unsafe or “cruel” [30]. In addition, criticism is often raised about manualized treatments detracting from the therapeutic relationship [29, 31].

### Supportive counselling

Supportive counselling (SC), based on Rogerian principles and the traumagenic model [32], has been shown to lead to improvements in PTSD and depression symptoms in adolescents in the USA [27] and South Africa [6]. This treatment was chosen as a comparator treatment (to PE-A) as it is an active treatment with demonstrated success that does not have a trauma focus. SC sessions center around developing a trusting, empowering, and validating therapeutic relationship and adolescents decide on the agenda for the sessions. Nurses provide active listening, empathy, and encouragement to express feelings and reinforced self-efficacy. Non-directive problem solving and keeping a diary are skills taught to participants.

This nested, qualitative (empiricist framework) study compares the experiences and perceived efficacy of two PTSD interventions by treatment users (adolescents with PTSD) and treatment providers (supervised nurses). This is the third of 3 qualitative studies, the first describing the impediments and catalysts to task-shifting of the treatments experienced by school liaisons and the treating nurses [33], and the second describing stigma-related experiences of adolescent participants accessing psychotherapy for PTSD in their school-based setting [34].

## Methodology

### Setting

For the RCT, trauma-exposed adolescents (aged 13 to 18) with PTSD were recruited at their respective schools from lower income high schools around Cape Town (South Africa) by presenting the treatment study and explaining the diagnosis of PTSD to students. Interventionists were registered nurses from outside of the community where the schools were situated, who were studying towards a diploma in psychiatry at Stellenbosch University. They were randomly assigned within a permuted block design to provide both treatments to prevent therapist confound. They were trained and supervised in both PE-A and SC protocols [3]. Nurses attended three days of training in PE-A from two authors (JR and EY) both of whom are experienced in implementing and training of the protocol. Training included theoretical and practical components such as reviewing of case studies and role plays. One author (JR) experienced in SC principles provided a one day training and supervised a further 16 hours of peer roleplay of the treatment components. Field workers included 2 recruiters and 2 independent assessors blind to the treatments.

### Participants

This qualitative study is nested within the main RCT study [35]; Pan African Clinical Trials Registry: PACTR201511001345372). Due to limited resources, only adolescents and interventionists who were entered inot the RCT in the first year (2014) were asked to participate. In 2014, 53 adolescents were screened for eligibility for the RCT by independent evaluators, using a structured interview, and 12 met inclusion criteria and were randomised to receive either PE-A or SC. These adolescents were purposively recruited by telephone during 2015, after having completed all post-treatment up and follow-assessments. All adolescents who had participated in the trial during the course of 2014 were invited, regardless of treatment arm or completion status, in an attempt to draw on a broad range of experiences. Ten adolescents agreed to participate in face-to-face, in-depth, interviews. Of the 10 participants, 8 also took part in follow-up focus groups. Two focus groups (4 in each group) were conducted, with each focusing on the type of intervention the adolescents received (PE-A or SC). Six nurses, previously psychotherapy-naïve, volunteered for the RCT and received training in both PE-A and SC and provided both treatments at 4 lower income high schools in Cape Town during 2014. The 6 nurses were purposively recruited by telephone during 2015 for this qualitative study. Three of the nurses consented to participate in individual interviews.

### Research instruments

The first author (who was not involved in the RCT) and a research assistant conducted recorded interviews and focus groups during 2015 as follows:

The first author conducted individual face- to- face semi-structured interviews (40–90 minutes) in English or Afrikaans. The discussion schedule focused onparticipants’ previous experiences with counselling, receiving treatment in the school setting, the counselling relationship, usefulness of the intervention, volunteering for the study, and recommendations for future interventions. Adolescents were also encouraged to participate in an English focus group (60 minutes) with the primary purpose of achieving data saturation. The interviewer was blinded to treatment arm for the individual interviews but unblinded for the focus group discussions, which were treatment specific. Interviews took place at the adolescents’ school or at Stellenbosch University’s Health Sciences Campus according to what best suited participants, while focus groups took place at the Health Sciences Campus with a co-facilitator (female fieldworker familiar to participants from Afrikaans middle schools).

Nurse participants did not arrive for the focus groups, so individual semi-structured interviews (60–90 minutes) were conducted. One interview was conducted via Skype as the nurse had returned to her hometown upon completion of her studies. No follow up interviews were conducted, however, nurse transcripts were emailed to participants to confirm the accuracy of the transcripts.

### Analysis

Fifteen audio recordings were transcribed by the first author and a research assistant (3 nurse interviews, 10 adolescent interviews, 1 adolescent SC focus group, 1 adolescent PE-A focus group). The first 2 authors read through the transcripts to identify emerging themes. The first author did thematic content analysis using Atlas.ti software to assist with coding the transcripts. After analysis, the second author re-read the transcripts and included any contradicting and/or outstanding data.

This article focuses on two richly described themes: (1) perceptions of the intervention and (2) usefulness of the intervention. Other themes identified have been described elsewhere [33, 34].

### Ethics

Stellenbosch University Human Research Ethics Committee approved the study (N12/06/031) and the Western Cape Education Department gave permission for the study to be conducted at the schools. Nurses and parents provided written informed consent and adolescents provided assent. Transcripts were de-identified and stored with the audio- recordings in a locked research office. Participants received reimbursement for their time and transport costs (up to ZAR 75 = US$ 5.45 for adolescents who participated in both the focus group and individual interview and ZAR 50 = US$ 3.36 grocery voucher for the nurses).

## Results

### Participants

The two adolescents who declined participation were both female and from the SC arm aged 16 (Afrikaans) and 17 (English). Two of the three female nurses who declined participation were Afrikaans speaking, the other was English. Table 1 provides demographic details (with pseudonyms) for consenting participants. For easy reference, pseudonyms are gender specific and start with “N” (nurse), “S” (SC adolescent) or “P” (PE-A adolescent). The last letter indicates if the participant was English, Afrikaans, or Xhosa speaking (ending on letter “e”, “a”, or other respectively).

**Table 1:** Participant pseudonyms and demographics.

Pseudonym	Home language	Gender	Consent	Age	Arm
‘Natasha’	Afrikaans	F	IDI & FG	N/A^a^	Nurse
‘Natalia’	Afrikaans	F	IDI & FG	N/A	Nurse
‘Noleen’	Xhosa	F	IDI & FG	N/A	Nurse
‘Paula’^b^	Afrikaans	F	IDI^c^ & FG^d^	15	PE-A^e^
‘Phoebe’	English	F	IDI only	15	PE-A
‘Precious’	Xhosa	F	IDI & FG	19	PE-A
‘Princess’^f^	Xhosa	F	IDI & FG	19	PE-A
‘Patrick’	Xhosa	M	IDI & FG	19	PE-A
‘Susanna’	Afrikaans	F	IDI only	14	SC^g^
‘Sonja’	Afrikaans	F	IDI & FG	15	SC
‘Sara’	Afrikaans	F	IDI & FG	16	SC
‘Seuna’	Afrikaans	M	IDI & FG	15	SC
‘Siyoli’	Xhosa	F	IDI & FG	19	SC

^a^ N/A: data not applicable

^b^ ‘Paula’ is the only participant who prematurely terminated her treatment (after 5 sessions)

^c^ PE-A: prolonged exposure therapy for adolescents treatment arm

^d^ IDI: in-depth interview

^e^ FG: focus group

^f^ ‘Princess’ did not arrive for the focus group because of transport limitations

^g^ SC: supportive counselling treatment arm

### Experience with treatment

Note that participants used the term ‘counselling’ to describe both treatment interventions. In an effort to authentically represent responses, the term is used here as said but should not to be confused as a reference to SC only.

Counselling was different from what participants expected. ‘Precious’ anticipated it would be similar to counselling seen on TV and ‘Seuna’ expected the counselling to work faster, like going to the doctor and receiving a tablet. ‘Princess’ and ‘Siyoli’ reported initial uncertainty about confidentiality. Nevertheless, participants experienced great relief in the relationships they forged with nurse counsellors and fieldworkers alike, saying that the fieldworker “always had a smile for me” (‘Seuna’). Similarly ‘Patrick’ said: “[The counsellor] is one of the people who is important in my life. And also [the fieldworker]… They play a very, very huge role in my life.”

### Advantages and disadvantages of SC

Nurses mentioned a number of advantages of the SC intervention, including working without a time restriction: “[the child] can share, even if it is just for 5 or 10 minutes” (‘Natalia’), and giving the child “power” and the counsellor “freedom” to work on any issue (‘Natasha’). This was echoed by ‘Sara’ who found the counselling helpful in addressing the death of her father and academic stress.

One disadvantage of this flexibility is described by ‘Natasha’:

“You did not know each day what this one’s reaction will be or what response you will get out of this one… Or you must find the right words to get the reaction or just get the one to say something. It made me kind of anxious.”

In the SC focus group, adolescents described what they did in counselling sessions: play games (e.g. dominoes or cards), write poetry, or draw. Although the games were not central to the treatment strategy (but rather a communicative aid), they were repeatedly highlighted by participants. ‘Sonja’ experienced counselling as more “fun” than expected, and assumed that the games were a distraction technique to “take your mind out of [pause] what happened.”

‘Seuna’ and ‘Sonja’ noted the burden of the paperwork they needed to complete (e.g. symptom self-report assessments). ‘Seuna’ compared it to an exam at school which left his brain tired. However, both participants suggested that they were willing to invest in some unpleasant tasks to get better.‘Sonja’ emphasized her eagerness to engage in the focus group because she felt it would be a forum to share her experiences and to get some sense of normalization.

### Advantages and disadvantages of PE-A

Nurses provided mixed reports on delivering PE-A. On the one hand, it was “easier to do” (‘Natasha’) because the intervention manual described each task every step of the way, but due to the perceived manual complexity it was “kind of hard to master” (‘Natalia’). It required a lot more preparation to become familiar with each session’s content, including attempts to “ask the questions from memory” (‘Natalia’) to avoid the children thinking that “you are not listening to them” (‘Noleen’) because of reading questions to them from a manual.

Compared to adolescents who received SC, adolescents who received PE-A provided more descriptive explanations of what took place in their sessions, reflecting on many components central to PE-A, including the use of metaphors, breathing retraining, imaginal exposure, and in-vivo exposure.

#### “She told me stories” (‘Patrick’)

Participants emphasized that the counsellors explained how the activities would help through the use of metaphors and never “forced” them to do activities.

She told me [the] story [about] the boy who was at the beach with the family… I think he lost the ball and the ball got inside the water. While he [went] to the water he was running, the water swept him down. And he has his mother … telling him a lot what he must do… It was stories like that. But I am not quite sure what the story was about.

(‘Patrick’)

‘Patrick’ was reflecting on the metaphor describing the importance of facing your fears used as part of the basic rationale for prolonged exposure therapy and although he did not remember exactly what the story was about, he identified it as helpful.

#### “How to breathe when the memory comes” (‘Precious’)

Breathing retraining enhanced a sense of competence and agency which was accessible beyond the treatment sessions to assist with public speaking (‘Patrick’), conflict (‘Phoebe’), exams (‘Precious’), and stress (‘Paula’). ‘Phoebe’ described the exercise:

“When you get on your nerves you just calm down, [voice drops lower and softer and slower] count to 10 and every time you count to 3… you say breathe to yourself. You say calm… and then just count further until you get till 10…

#### “I will close my eyes and recall everything” (‘Precious’)

Some participants initially resisted imaginal exposure “because that feeling was still with me. Just to talk or think about it was too much” (‘Paula’) and I would rather “push it to the back of my head” (‘Precious’). ‘Patrick’ realized that he needs help and this motivated him to engage in the activity although “it wasn’t easy.” ‘Natasha’ felt irritated with her participants when they rolled their eyes or complained about having to do the imaginal exposure “again”.

Regardless of the frustration and initial dislike of the method, it was applauded by the participants for being the most helpful part of their counselling journey. After many repetitions “I forget about the problem and feel happy” (‘Patrick’) and “It started being a norm in my mind; I started learning how to handle it when it comes” (‘Precious’). Part of these repeated exposures included a homework activity where participants had to listen to recordings of their sessions:

“Oooo [emphasis], the recording activity. I hated it. You had to listen to the recorder and then write it. How long did it take, or what were you feeling at that moment, you had to write it down on a paper. You had to do it at home. It was homework. I hated it!”

(‘Precious’)

#### In-vivo exposure

When asked what the most difficult things were that her counsellor asked her to do, ‘Phoebe’ had no trouble listing five real life experiments and the use of the subjective units of distress scale to deal with these. She was the only participant to describe in vivo exposure homework.

“Standing in a group of people I don’t know; being around cats; swimming around boys; um, speaking to people I like, just met; and doing activities with a lot of people… I had to spend a few minutes every day… I have to score myself, out of 10 how am I feeling and then I calm down. It’s like how can I grow, and then I have to like adjust which ones was good.”

(‘Phoebe’)

### Experience of the therapeutic relationship

Regardless of treatment modality, participants listed the counselling relationship as the number one most important component of their experience. They described the counsellors as accepting, maternal, and trustworthy. Many participants highlighted that they would contact the fieldworkers and counsellors in the future should a friend be in need of some help.

### “Someone you can trust” (‘Princess’)

‘Susanna’ decided to trust the counsellor because of “the way she spoke and … that she understood everything I said,” while ‘Patrick’ said it was because “she will give me facts” and “she would tell me more about what I don’t understand.” It was important that the counsellor showed interest (‘Princess’, ‘Siyoli’) as demonstrated in the nurse counsellors’ voluntary (unpaid) service. This trust enabled participants to be honest even when they were “not honest with my mother” (‘Paula’).

#### Respectful and kind

PE-A participants described their counsellor as “polite… kind” (‘Precious’), “friendly” (‘Patrick’), and “tolerant… very calm” (‘Precious’). ‘Paula’ “knew I can trust her… she would not judge me or relay it.” ‘Siyoli’ said that the counsellor demonstrated respect “even if I am a kid.” ‘Phoebe’ was relieved that “somebody believed me.” The counsellor motivated ‘Patrick’ to “overcome life challenges.” ‘Precious’ valued the fact that her counsellor would sometimes cut a session short when the participant did not feel like she could carry on: “If she was very different from the way she was I wouldn’t have finished the research, I would just have run away” (‘Precious’).

#### Maternal

‘Patrick’ and ‘Siyoli’ described their counsellors as mothers during their individual interviews. This was echoed in the PE-A focus group. Characteristics of this maternal relationship included the counsellor being available, even if it was just by telephone (‘Siyoli’), feeling like they had known each other for a long time (‘Precious’), teaching the participant about life (‘Paula’), and being friendly (‘Patrick’). Furthermore, ‘Patrick’ believed the feeling was mutual: “I saw my mother and she saw her son.”

#### Unique relationship

The therapeutic relationship stood in stark contrast to some of the participants’ existing relationships with their families and teachers. Apart from the counsellors’ interpersonal skills, this difference in experience was also rooted in the fact that these counsellors were part of the adolescents immediate community.

Ok, I didn’t trust her at first but I have to, I have to tell my counsellor. But she tells me everything about her then I see that “no, I can trust this person” because she’s not my friend… not part of my family but I know that I can tell someone… It is not easy to talk about other people’s situation or problem because if I tell someone that I know or I live with, it will end up that everybody will know my problem… It is easy to trust that person [outside of the community] because you know that she or he can’t tell anyone. Because you told him or her your problem and you trust that person that I know this person. He can’t or she can’t tell anyone. Because if I tell some of my family, when she shout at me, she will mention my problem and it will hurt me so much when he reminds me back and all the memories will come back.

(‘Princess’)

### Navigating therapeutic boundaries

Nurses described how their relationships with the children developed, sometimes leading to boundary considerations. Both ‘Noleen’ and ‘Natalia’ found birthdays exceptionally challenging, wondering if they could not just give a chocolate as a present but “we were not allowed to do that… you must rather not do it” (‘Natalia’). ‘Natasha’ described that simply wishing “them good luck with the exam it meant a lot to them. Just that little extra attention that they received.”

Participants had access to the counsellors’ cellphone numbers. A while after ‘Natasha’ had completed providing the PE-A treatment, one of the participants sent her a text message indicating suicidal intent. ‘Natasha’ was able to refer the participant to receive appropriate help. This was not the only difficulty in terminating the therapeutic relationship as ‘Noleen’ described:

I might be the only person that can do something… That awareness and that understanding ja, it just, it helped me … to be in the process and forget about my own um… other things that I was supposed to do … As much as some of the children had difficulty terminating with me and I also had difficulty terminating with them …. Because it was like, I’m here, I came and then I disappear. And you also find that there are people in their life who were there and then disappeared. So I was like, “what, what system is there?” You know, I would often ask about that. This child from me where is she or he going to go.

(‘Noleen’)

### Experience of treatment effectiveness

Adolescents were asked to reflect on their lives before and after the intervention. During the PE-A focus group, there was a powerful non-verbal emotional shift best described as a transition from upbeat camaraderie to quiet reflection. ‘Precious’ described it as “You asked me what does it feel like to be this kind of person that I am today. And I remember where I come from.” ‘Natasha’ and ‘Natalia’ also reflected on the feedback they received from other participants and their teachers to motivate them and make the most benefit of the treatment.

### Perception of the impact of PTSD symptomson functioning before and after the-interventions

The rich data reflecting adolescents’ perceptions of the benefits of the intervention is best summarized in light of the following powerful description by ‘Precious’ of her experience with PTSD. This quote formed the basis of findings illustrating whether participants overcame these symptoms.

Well, I was very difficult child. Difficult to deal with because I had that state of mind that nobody would understand me. Nobody would know me. So I was in that blocking space. So I didn’t talk to anyone, didn’t do anything. I started failing and I started having that state of mind that I don’t want anyone to know what I am going through or to think that I’ll be able to talk to anyone. I just wanted to be alone. I didn’t want to think about it. I didn’t want to know how to solve it. I just wanted it to go away. Put it at the back of my mind. But actually at the end it did happen. Because it always came back and always haunting me. I was failing. It was going to affect me and everybody else in my life because I was distant also to my family. It was a horrible time. I just wanted to be alone, wanted to be in my own space. No one could bother me or anything. Now talking to someone in that space: I didn’t want to. Even if someone would ask me, I just blow out in anger. Shout at them. So I didn’t want to get help.

(‘Precious’)

#### Impact on academic performance

‘Sonja’ reported concentration difficulties that impaired academic performance while ‘Sara’ disclosed that rumors about her were affecting her school work. With the help of counselling, ‘Sara’ indicated that she doesn’t “care what they think anymore.” ‘Precious’ found the intervention improved her academic performance:

I am a new person. Very very new person. The person that’s the same person I was. The active, talkative, very annoying girl. I am still… unleashing that self of mine. I am still busy with that person. But the counselling has really really helped me. Because now I am able to be a better person. I passed my grade 11 with enormous marks… I am now going through my grade 12 with a very very good self-esteem. Because if I was still that person, I would never be going through this grade 12. Ja, so it really really did help me.

#### Moving from secrecy and withdrawal to re-integration

‘Phoebe’ said that before the counselling she was “short tempered,” “edgy,” and “distrustful.” She found it difficult to talk about the traumatic event and had not yet disclosed the event to her parents: “I tried to keep it for myself. Nobody, I thought nobody needed to know. It will get better but it just made me go insane, not better. [Laughs].” Since her parents had to sign consent, the fieldworker helped her to disclose her secret to her parents. Although it is still hard, she is “kind of getting used to speaking about it now.” In addition, the therapy “helped [her] to trust more” and improve her anger management skills.

‘Susanna’ explained that after the therapy she was “calmer” and could “spend time at home relaxing” leading to improved quality of interactions with her friends. Similarly, ‘Patrick’ described how he used to withdraw from his friends, preferring to “sleep at home” which made him feel lonely. He also reported that he was involved in fighting, making the “wrong decisions” and having “no respect for other peoples.” When probed, he explained that he was hurting so much, that he felt entitled to hurt others. This changed after participating in therapy:

Now I am feeling happy and free… everything is alright now… I have a good relationship with friends and my classmates. I knew what was wrong and what was right… And wherever I go you cannot hear ‘‘Patrick’ is in a fight.’ No. You will never hear that.

‘Siyoli’ reported ongoing social challenges. Before receiving counselling she did not want to go to school and felt very lonely because nobody wanted to speak to her. This continued after therapy where “I try to be with people, but no one. No one. Not even my cousin. They just ignore me” (‘Siyoli’).

#### Coping with symptoms

Before receiving therapy, participants used distraction techniques. ‘Paula’ “tried to forget about it, but then every time then it just comes up and then I feel down again.” As she is a very private person, outsiders would not have been able to see that those thoughts were haunting her. ‘Princess’ experienced emotional distress when she came close to the perpetrator, a close family member: “Everything come in my mind and it made me cry.” She had a particularly challenging journey learning that if she has “a grudge for someone it can mess my future… if I hold a grudge my future will fall down.” She described how she decided to forgive the perpetrator although she no longer trusts him.

Some participants admitted to more extreme avoidance behaviour, such as running away (‘Siyoli’, ‘Sonja’) and thoughts of suicide (‘Siyoli’, ‘Princess’), in their attempts to escape the painful symptoms.

‘Sara’ and ‘Seuna’ reported sleep difficulties which required that they sleep with the light on and door open. After therapy these symptoms improved and ‘Seuna’ “is not scared anymore.”

‘Paula’ prematurely terminated PE-A treatment and felt frustrated that she still keeps things to herself. Similarly, although ‘Siyoli’ found therapy beneficial, she continues to experience intrusive thoughts at the same frequency as before:

I forget everything we talking about. Everything. All that memories. I just forget everything. Even at home I just ignore them. If they shout, I just be happy. Always happy. I always tell myself that … I still have that time where I lose my mind. Even if I am concentrating to something, my mind just go away and stop. Then when it comes back, it just go back to old things. To 2010 accident. And then I will stand there and shake. And then I will cry. Even if my mind come back, my mom will tell me, I will say it was not happening. No you are lying. I don’t know what is happening to me. I can’t feel it. It just happened.

### Treatment effectiveness vs transferability

‘Precious’ explained that she is still on a journey despite symptom improvement: “I’m still unleashing that self of mine. I am still busy with that person. But the counselling has really, really helped me.”

‘Sara’ described to others in the SC focus group that she sometimes does not “want to think about it. Then it come and then I do something that don’t remind me of that.” Thereafter, group members admitted that intrusive thoughts occasionally recurred after treatment, making them feel “horrible… unhappy” (‘Sonja’), “very sad” (‘Sara’), and “makes you cry” (‘Siyoli’).

Throughout the individual interview, ‘Sonja’ seemed objectively depressed. She described how classroom conversations about divorce, drugs, absent parents “puts things on [her]” which makes her cry. She uses distraction as a coping mechanism.

‘Siyoli’s’ individual interview suggested that it was not the counselling that enabled her to change, but rather the birth of her child which made her stay at home and try and rebuild a new life. She admitted that, “even now, it is very difficult but not the way it was very difficult… Sometimes come to my mind I must go, leave this baby here. Huh-uh. I must stay at home.”

Overall, adolescents in the SC group perceived distraction as a helpful technique which they continued to apply after treatment termination in the form of “play[ing] with children” (‘Sonja’ & ‘Sara’), “play[ing] with my baby” (‘Siyoli’), “play[ing] with my dog (‘Seuna’) or “cleaning the house” (‘Sara’).

From the nurses’ perspective, SC was more transferable than PE-A. ‘Natalia’ preferred SC because it was better suited to her personality while ‘Natasha’ often implemented the SC skills in her current occupation because of time restraints:

Patients heap up and there are 30 patients on a day that you need to see… there is no time to do the prolonged exposure, not really, but the supportive counselling you can do … Just so that they know there is somebody that listens, there is help, there is somebody you can go to for help. That already makes a big difference.”

‘Noleen’ was thrilled to transfer her newly acquired counselling skills at a private psychiatric facility to engage with an unruly intoxicated patient. “I just use my reflection skill, I just validate her and do all that and slowly the patient was still like, nagging and nagging, but the most important thing is that she was walking [laughing]” (‘Noleen’). She emphasized that if she was able to “memorize” the treatment and had the power to influence her superiors, she would insist on implementing PE-A in her workplace as she experienced it as the most effective treatment.

## Discussion

The adolescents in this study clearly highlighted subjective differences between PE-A and SC. Whilst the description of SC was vague, participants in PE-A provided a clear account of homework and exposure activities. Both PE-A and SC participants continued to use the techniques acquired during sessions: PE-A participants used breathing retraining for exams, public speaking, and anger management; SC participants applied distraction techniques (such as playing with children or pets, or cleaning the house). Both PE-A and SC participants reported benefitting from the intervention they received. PE-A participants referred to their posttreatment process as a journey which equipped them to deal with challenges. The reports from PE-A participants seemed to indicate that they experienced a reduction in reexperiencing, whereas SC participants reported residual reexperiencing, depressive and anxiety symptoms.

The centrality of the therapeutic relationship to the success of both interventions, described by nurses and participants, emphasise the importance of that relationship and challenges the criticism often voiced about manualized treatment being too technique driven [26]. Both PE-A and SC participants described the counsellors as accepting, maternal, and trustworthy. The nurses in this study expressed some concern that the adolescents would perceive them as uncaring and rigid for using the PE-A manual. However, their concerns were not supported by the feedback from the PE-A participants.

The nurses perceived both treatments as beneficial. SC was deemed to be most transferable within the existing time and institutional constraints they would return to. The nurses described the PE-A intervention as being more difficult to implement. This observation should be viewed within the context of their reporting on the first few cases they had treated using the PE-A intervention and it is likely that with more experience they would become more comfortable with the content and could implement it within their community healthcare clinics. These preliminary findings contribute to the growing body of literature describing the effectiveness of established evidence-based mental health treatments provided through task-shifting [36].

Several limitations of this study merit consideration. Only 10 of the 12 participants and 3 of the 6 nurses from the first year of the RCT study participated in this qualitative investigation. This limits the sample size and, consequently, limits generalizability of these findings. Qualitative studies, however, enrich quantitative research with more individual perspectives of the participating stakeholders and help inform planning further implementation of interventions, taking their perspectives into consideration. Generalisabillity across gender was limited by the low number of males in the study.

## Conclusion

Despite these limitations, the study provides rich and nuanced insights into the experiences of adolescents accessing PTSD treatments through task-shifting in their community school setting. as well as into the experiences of the newly trainged nurses providing the interventions. Taking into account the small sample size, some interesting qualitative observations can be made:

Adolescents in both PE-A and SC treatments delivered by non-specialist health workers described the therapeutic relationship as warm and the counsellors as accepting, maternal, and trustworthy. Whilst all participants reported symptom reduction and continued to use the tools after treatment, adolescents who received SC reported ongoing distressing reliving their experiences.

The nurses perceived both treatments as beneficial but described SC as more easily implementable within their work environments. They reported that the central skills utilized in SC, namely empathic responding and non-directive problem solving, could be implemented within their work routine. Although PE-A was described as a more effective intervention for PTSD, it was found to require more time to prepare for and some doubts were expressed about their ability to implement PE-A without institutional and policy support when returning to their places of work.

The debate is ongoing on whether the implementation of evidence-based treatments in LMICs is effective, transportable, scalable, culturally sensitive, and financially sustainable. There is, however, a growing number of RCTs demonstrating the effectiveness of EBTs implemented in a task-sharing paradigm [37].

It is important that the perspectives of those utilizing as well as providing the services are heard and taken into consideration when planning and implementing mental health interventions within communities.
